# Comparative study of alcohol use, alcohol use disorder, and consequences among young people and adults with injuries in Northern Tanzania

**DOI:** 10.1016/j.afjem.2026.100975

**Published:** 2026-05-01

**Authors:** Winfrida C. Mwita, Elizabeth M. Keating, Rune Nathaniel Philemon, Baraka Moshi, Doreen O. Msemakweli, Alice Andongolile, Florida J. Muro, Blandina T. Mmbaga, João Ricardo Nickenig Vissoci, Sia E. Msuya, Catherine A. Staton

**Affiliations:** a School of Public Health, Department of Epidemiology and Applied Biostatistics, KCMC University, Moshi, Tanzania; b Kilimanjaro Clinical Research Institute, Moshi, Tanzania; c Division of Pediatric Emergency Medicine, University of Utah, Salt Lake City, UT, USA; d Department of Pediatrics and Child Health, Kilimanjaro Christian Medical Centre (KCMC), Moshi, Tanzania; e Duke Global Health Institute, Duke University, Durham, NC, USA; f Department of Paediatric and Child Health, School of Medicine, KCMC University, Moshi, Tanzania; g Community Health Department, Kilimanjaro Christian Medical Centre, Moshi, Tanzania; h Department of Emergency Medicine, Duke University School of Medicine, Durham, NC, USA; i Global Emergency Medicine Innovation and Implementation (GEMINI) Research Center, Duke University, Durham, NC, USA

**Keywords:** Alcohol use, Alcohol use disorder, Injury, Emergency department, Young people, Tanzania

## Abstract

**Introduction:**

Alcohol use is a major risk factor for injury, a leading cause of morbidity and mortality among young people in sub-Saharan Africa. In Tanzania, high rates of alcohol use disorders and heavy episodic drinking have been documented. However, age differences in alcohol use and related harm among injury patients are poorly described. Understanding these differences is important for informing targeted interventions.

**Methods:**

We conducted a cross-sectional analysis using linked data from the Kilimanjaro Christian Medical Centre Trauma Registry and the Pragmatic Randomized Adaptive Clinical Trial (2020–2025). Acute injury patients aged ≥18 years presenting within 24 h were included. Alcohol use was assessed using the Alcohol Use Disorders Identification Test, and consequences using the Drinker Inventory of Consequences. Outcomes were compared between young people (18–24 years) and adults (≥25 years) using descriptive, age-stratified analyses.

**Results:**

Among 2427 injury patients (520 young people; 1907 adults), current alcohol use was reported by 46.5% of young people and 59.4% of adults. Adults more often reported alcohol use in the past four weeks (28.7% vs. 18.9%) and within six hours prior to injury (21.3% vs. 11.4%), and were more likely to drink ≥4 times per week (14.1% vs. 5.8%; *p* < 0.001). Young people more frequently reported injury to themselves or others due to drinking in the past year (14.0% vs. 9.4%; *p* = 0.026) and higher physical alcohol-related consequences (85.7% vs. 73.5%; *p* = 0.010). The prevalence of alcohol use disorder was similar between groups (37.2% vs. 39.6%).

**Conclusion:**

Although adults reported more frequent alcohol consumption, young people had comparable prevalence of alcohol use disorder and experienced greater alcohol-related physical consequences, including injury. These findings highlight distinct age-related drinking patterns and a disproportionate burden of harm, suggesting the need for age-responsive alcohol screening and intervention strategies within emergency care settings.

## African relevance

• Alcohol use is a major contributor to injuries among patients presenting to emergency departments in sub-Saharan Africa.
• Among injured patients, alcohol use and alcohol-related harm differ between young people and adults.
• Greater alcohol-related harm is observed among young people despite lower drinking frequency.
• This underscores the need for age-responsive alcohol screening and care in emergency settings.

## Introduction

Alcohol use remains a global public health problem due to its substantial contribution to morbidity, mortality, and social harm. The World Health Organization (WHO) estimates that harmful alcohol use was responsible for 2.6 million deaths globally in 2019 and contributed to 115.9 million disability-adjusted life years (DALYs), with injuries accounting for over 27.6% of alcohol-attributable deaths [1]. The burden is particularly pronounced in low- and middle-income countries (LMICs), especially in sub-Saharan Africa (SSA), where the age-standardized rate of alcohol-attributable deaths is among the highest globally [1] Young people (ages 15–24) are disproportionately affected, with alcohol being the leading contributor to injury, violence, and early mortality in this age group [1,2].

In Tanzania, alcohol-related harm is a growing concern. Studies document high rates of alcohol consumption and alcohol use disorders (AUD), especially among young men. In Northern Tanzania, 10.5% of individuals aged 15–24 screened positive for AUD [3]. In clinical settings, alcohol use is also prominent among injury patients; a referral hospital found that 30% of acute injury patients tested positive for alcohol via breathalyzer on arrival to the emergency department [4]. Despite this burden, alcohol regulation and treatment services in Tanzania remain fragmented, with limited integration of routine screening and brief intervention into healthcare settings [5]. Little is known about how these alcohol-related risks and patterns differ by age among injury patients, for whom alcohol is a major proximal risk factor for both unintentional and intentional injuries [6,7].

Age plays an important role in shaping alcohol use behaviors and consequences. Drinking patterns vary by age and are shaped by social, cultural, and developmental factors, with younger individuals more likely to engage in episodic heavy drinking and adults tending toward more routine, habitual consumption [2,8,9]. These differences are not only behavioral but also neurobiological; exposure to alcohol during adolescence and young adulthood, a period of ongoing brain development, can alter neural pathways related to impulse control and reward processing, [[10], [11], [12]] thereby increasing vulnerability to hazardous drinking and the subsequent development of AUD [13]. Early initiation of alcohol use has also been linked to genetic predisposition for addiction and worsened mental health outcomes [14].

However, evidence on age-specific drinking patterns among Tanzanian injury patients remains scarce. While some recent studies have examined subgroups, such as gender differences among emergency department patients, [15] most continue to aggregate across age groups or focus on the general population. Alcohol-related injuries place additional strain on emergency services, where screening and treatment for AUD remain limited. Generating age-specific evidence is therefore important to inform targeted screening and intervention strategies within emergency departments. This study addresses this gap by comparing alcohol use and alcohol-related consequences between young people and adults presenting with injuries in Northern Tanzania to inform age-specific alcohol interventions for injury patients.

## Methods

### Study design and setting

This cross-sectional secondary analysis was conducted at Kilimanjaro Christian Medical Centre (KCMC), a tertiary referral hospital in Moshi, Northern Tanzania. KCMC serves as the regional referral hospital for the Northern Zone, and its Emergency Department (ED) manages approximately 2000 injury cases annually [16]. Ethical approval for this secondary analysis was obtained from the KCMC University Ethics Committee (Certificate #2708) and the Tanzanian National Institute of Medical Research (NIMR/HQ/R.8a/Vol.IX/4839).

### Data source

This analysis used two linked data sources: the KCMC Trauma Registry and the KCMC Pragmatic Randomized Adaptive Clinical Trial (PRACT) Registry. The Trauma Registry prospectively collects routine clinical and demographic data for all patients aged ≥18 years presenting to the ED with acute injuries. It includes information on demographics, injury type and mechanism, vital signs, clinical care, and ED outcomes. Patients presenting for follow-up care or those who die prior to evaluation are excluded. The PRACT trial is a randomized controlled trial evaluating a brief alcohol intervention among injury patients. It provided additional research-specific data, including self-reported alcohol use prior to injury, Alcohol Use Disorders Identification Test (AUDIT) scores, breathalyser results (blood alcohol concentration >0.0 g/dL), and Drinker Inventory of Consequences (DrInC) scores. Data were collected after participants were clinically sober and provided informed consent. The trial is registered at ClinicalTrials.gov (NCT04535011). Detailed trial methodology and ethical approvals have been published elsewhere [17]. Data included in this analysis were collected between October 2020 and February 2025.

### Study population

This study included injury patients aged ≥18 years presenting to KCMC’s ED within 24 h of injury. Only clinically sober patients who provided informed consent were included. Participants were categorised as young people (18–24 years) and adults (≥25 years), consistent with prior alcohol research and global public health definitions [3,9,[18], [19], [20]].

### Measures

Sociodemographic information (age, sex, tribe, religion, education, employment status, and monthly income) was obtained from the Trauma Registry. Alcohol use data were obtained from the PRACT trial using structured questionnaires and the Timeline Follow back (TLFB) method, [21] a calendar-based, interviewer-administered tool designed to enhance recall of substance use over a specified period. Alcohol use prevalence was assessed for the past 12 months, past 28 days, and within 6 h prior to injury.

Alcohol use disorder screening was conducted using the World Health Organization Alcohol Use Disorders Identification Test (AUDIT) among current drinkers (past 12 months) [22]. An AUDIT score ≥8 indicated hazardous or harmful alcohol use or probable alcohol use disorder. The AUDIT has been validated in Tanzanian injury populations [23]. Among participants reporting alcohol use in the month prior to injury, drinking frequency and quantity were assessed using AUDIT items. One standard drink was defined as containing 10 g of ethanol [24].

Alcohol-related consequences were assessed using the Drinker Inventory of Consequences (DrInC) [25]. For this analysis, past 3-month consequences across physical, interpersonal, social responsibility, impulse control, and intrapersonal domains were examined. The physical subscale reflects adverse physical states resulting from excessive drinking, such as hangovers, sleep disturbances, vomiting, impaired physical health or appearance, and alcohol-related injury, while other subscales capture consequences related to relationships, role functioning, impulsive behaviours, and internal emotional states.

### Statistical analysis

Data analysis was conducted using Stata version 15 (StataCorp LLC, College Station, TX, USA) [26]. Descriptive statistics summarized participant characteristics, alcohol use measures, and alcohol-related consequences, stratified by age group (18–24 years vs. ≥25 years).

Categorical variables were summarized using frequencies and proportions, and compared using Pearson’s chi-square or Fisher’s exact tests, as appropriate. Continuous variables were summarized using means (standard deviation) or medians (interquartile range) and compared using independent *t*-tests or Mann–Whitney U tests, depending on distribution. Statistical significance was set at *p* < 0.05.

## Results

### Socio-demographic characteristics of the study participants

A total of 2427 injury patients were included, of whom 520 (21.4%) were young people (18–24 years), and 1907 (78.6%) were adults (≥25 years). The majority were male (80.4%), with a higher proportion among young people compared to adults (86.0% vs. 78.8%; *p* < 0.001). Educational attainment differed by age group (*p* < 0.001), with young people having a higher proportion of secondary education completion (46.6% vs. 22.6%), while adults had a higher proportion with primary education as their highest level (52.1% vs. 33.3%). Median monthly income was lower among young people compared to adults (TZS 150,000 vs. 240,000; *p* < 0.001) (Table 1).

**Table 1:** Socio-demographic characteristics of injury patients in the northern zone of Tanzania by age group (N = 2427).

Variable	Overall, N = 2427	Young People (18–24 years), N = 520	Adults (≥25 years), N = 1907	*p*-value
**Age**				<0.001
Mean (SD)	37.3 (15.1)	21.5 (1.9)	41.7 (14.3)	
**Sex**				<0.001
Male	1950 (80.4)	447 (86.0)	1503 (78.8)	
Female	477 (19.6)	73 (14.0)	404 (21.2)	
**Tribe**				0.017
Chagga	1162 (48.1)	226 (43.7)	936 (49.3)	
Pare	341 (14.1)	68 (13.2)	273 (14.4)	
Other	911 (37.8)	223 (43.1)	688 (36.3)	
Missing/Refused	13/2427	3/520	10/1907	
**Religion**				0.070
Muslim	473 (19.6)	116 (22.3)	357 (18.8)	
Christian	1938 (80.1)	403 (77.7)	1535 (80.8)	
Other	8 (0.3)	0 (0.0)	8 (0.4)	
**Highest Educational Attainment**				<0.001
No formal education	42 (1.7)	3 (0.6)	39 (2.1)	
Primary	1161 (48.1)	173 (33.3)	988 (52.1)	
Secondary	671 (27.8)	242 (46.6)	429 (22.6)	
Vocational	145 (6.0)	28 (5.4)	117 (6.2)	
College/University	396 (16.4)	73 (14.1)	323 (17.0)	
Missing/Refused	12/2427	1/520	11/1907	
**Employment Status**				<0.001
Student	89 (3.7)	78 (15.1)	11 (0.6)	
Unemployed	85 (3.5)	23 (4.4)	62 (3.3)	
Employed	2237 (92.8)	417 (80.5)	1820 (96.1)	
Missing/Refused	16/2427	2/520	14/1907	
**Personal Income (TZS per month)**				<0.001
Median (IQR)	200,000 (100,000–400,000)	150,000 (50,000–300,000)	240,000 (100,000–450,000)	

#### Prevalence of alcohol use among young people and adult injury patients in Northern Tanzania

**Fig. 1:**
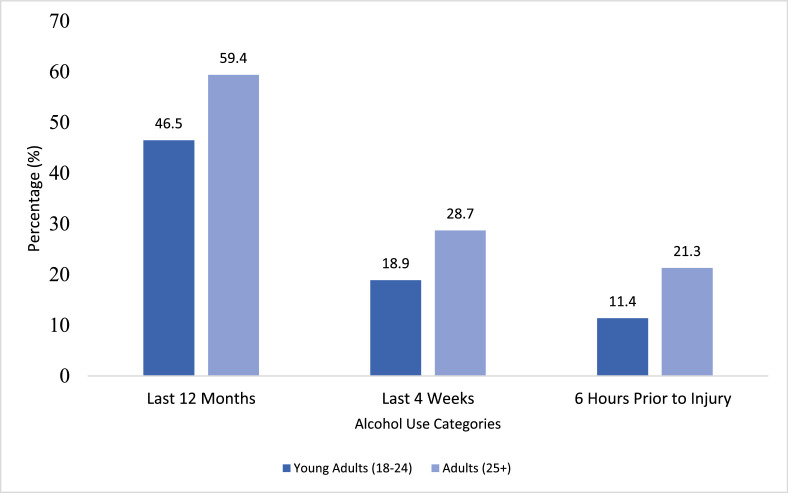
Reported alcohol use among injury patients by age group - young people (18–24 years, n = 520) vs Adults (≥25 years, n = 1907) *Percentages are based on available responses; Denominators vary slightly due to item non-response.*

Alcohol use was consistently higher among adults than young people across all timeframes (Fig. 1). This pattern was observed for current drinking (past 12 months), alcohol use in the past four weeks, and alcohol use within six hours prior to injury (*p* < 0.001 at each time point).

### Drinking risk levels and AUD among young people versus adults

Among current drinkers, most were classified as low-risk (AUDIT 1–7; 60.8%), with similar proportions among young people and adults. Distribution across higher AUDIT categories (8–15, 16–19, 20–40) was similar between groups, with no significant differences (*p* = 0.804). The prevalence of AUDIT ≥8 was comparable between young people (37.2%) and adults (39.6%) (*p* = 0.524) (Table 2).

**Table 2:** AUDIT drinking risk levels and AUD prevalence by age group among current drinkers (N = 1372).

AUDIT Drinking Risk Level	Total n (%)	Young People (n = 242) n (%)	Adults (n = 1130) n (%)	*p*-value
**AUDIT Score Categories**				
1–7	834 (60.8)	152 (62.8)	682 (60.4)	
8–15	324 (23.6)	56 (23.1)	268 (23.7)	
16–19	91 (6.6)	16 (6.6)	75 (6.6)	
20–40	123 (9.0)	18 (7.4)	105 (9.3)	
*Overall difference across scores*				0.804
**Total AUDIT ≥8 (AUD)**	538 (39.2)	90 (37.2)	448 (39.6)	0.524

*AUDIT-positive defined as score ≥8, indicating hazardous/harmful use or probable AUD. The AUDIT is a screening tool and does not provide a clinical diagnosis of AUD.

### Drinking pattern among injury patients

Among current drinkers, drinking frequency differed by age group (*p* < 0.001). Young people more often reported drinking monthly or less compared with adults (46.7% vs. 34.3%), whereas adults more frequently reported drinking four or more times per week (14.1% vs. 5.8%). Young people were also more likely to report that they or someone else had been injured due to their drinking in the past year compared with adults (14.0% vs. 9.4%; *p* = 0.026) (Table 3).

**Table 3:** Pattern of drinking behaviour among injury patients: comparison between young people and adults (N = 1372).

Question	Response Question	Young People (n = 242) n (%)	Adults (n = 1130) n (%)	*p*-value
1. How often do you have a drink containing alcohol?	Never	0 (0.0)	0 (0.0)	
	Monthly or less	113 (46.7)	387 (34.3)	
	2–4 times a month	57 (23.6)	286 (25.3)	
	2–3 times a week	58 (24.0)	298 (26.4)	
	4 or more times a week	14 (5.8)	159 (14.1)	<0.001
2. How many drinks containing alcohol do you have on a typical day when you are drinking	1–2	93 (38.4)	426 (37.7)	
	3–4	84 (34.7)	402 (35.6)	
	5–6	42 (17.4)	185 (16.4)	
	7–9	12 (5.0)	48 (4.3)	
	10 or more	11 (4.6)	69 (6.1)	0.870
3. How often do you have six or more drinks on one occasion?	Never	147 (60.7)	620 (55.0)	
	Less than monthly	45 (18.6)	228 (20.2)	
	Monthly	16 (6.6)	96 (8.5)	
	Weekly	29 (12.0)	134 (11.9)	
	Daily or almost daily	5 (2.1)	50 (4.4)	0.276
4. How often during the last year have you found that you were not able to stop drinking once you had started?	Never	118 (77.7)	841 (74.5)	
	Less than monthly	21 (8.7)	125 (11.1)	
	Monthly	12 (5.0)	56 (5.0)	
	Weekly	17 (7.0)	79 (7.0)	
	Daily or almost daily	4 (1.6)	28 (2.5)	0.749
5. How often during the last year have you failed to do what was normally expected of you because of drinking?	Never	213 (88.0)	961 (85.1)	
	Less than monthly	19 (7.9)	103 (9.1)	
	Monthly	3 (1.2)	38 (3.4)	
	Weekly	6 (2.5)	22 (2.0)	
	Daily or almost daily	1 (0.4)	5 (0.4)	0.427
6. How often during the last year have you needed a first drink in the morning to get yourself going after a heavy drinking session?	Never	214 (88.8)	956 (84.7)	
	Less than monthly	14 (5.8)	78 (6.9)	
	Monthly	6 (2.5)	27 (2.4)	
	Weekly	3 (1.2)	41 (3.6)	
	Daily or almost daily	4 (1.7)	27 (2.4)	0.314
7. How often during the last year have you had a feeling of guilt or remorse after drinking?	Never	148 (61.2)	677 (60.0)	
	Less than monthly	65 (26.9)	289 (25.6)	
	Monthly	11 (4.6)	63 (5.6)	
	Weekly	8 (3.3)	64 (5.7)	
	Daily or almost daily	10 (4.1)	35 (3.1)	0.504
8. How often during the last year have you been unable to remember what happened the night before because of your drinking?	Never	203 (83.9)	938 (83.1)	
	Less than monthly	31 (12.8)	113 (10.0)	
	Monthly	3 (1.2)	34 (3.0)	
	Weekly	3 (1.2)	38 (3.4)	
	Daily or almost daily	2 (0.8)	6 (0.5)	0.129
9. Have you or someone else been injured as a result of your drinking?	No	179 (81.0)	846 (81.7)	
	Yes, but not in the last year	11 (5.0)	92 (8.9)	
	Yes, in the last year	31 (14.0)	97 (9.4)	0.026

### Alcohol-Related consequences among injury patients

Among the current drinkers, 645 participants (98 young people; 547 adults) had data on alcohol related consequences. In this subset, physical consequences were more common among young people than adults (85.7% vs. 73.5%; *p* = 0.010).

No age differences were observed across DrInC subscales of social responsibility, interpersonal, intrapersonal, or impulse control consequences (*p* > 0.05 for all comparisons) (Table 4).

**Table 4:** Alcohol-related consequences (DrInC subscales) among young people and adults (N = 645).

Variable	Total (n = 645) n (%)	Young People (n = 98) n (%)	Adults (n = 547) n (%)	*p*-value
DrInC subscales				
Physical	486 (75.4)	84 (85.7)	402 (73.5)	0.010
Social responsibility	396 (61.4)	62 (63.3)	334 (61.1)	0.680
Interpersonal	407 (63.1)	67 (68.4)	340 (62.2)	0.241
Intrapersonal	428 (66.4)	66 (67.4)	362 (66.2)	0.822
Impulse control	455 (70.5)	73 (74.5)	382 (69.8)	0.352

Note: n (%); past-3-month consequence (≥1 item). DrInC subset (N = 645); denominators may vary due to item non-response.

## Discussion

To our knowledge, this is the first study in Tanzania to compare alcohol use patterns, AUD and alcohol-related consequences between young people and adults presenting with injuries. Three key findings emerged. First, although adults reported more frequent alcohol use, young people were significantly more likely to report that they or someone else had been injured as a result of their drinking within the past year. Second, AUD prevalence (AUDIT ≥8) was similarly high across age groups. Third, young people reported significantly more physical consequences of alcohol use, indicating a greater acute harm burden despite lower drinking frequency. These findings highlight age-specific risk patterns and the need for targeted prevention in Tanzania and similar LMIC settings.

Young people were significantly more likely to report that they or someone else had been injured as a result of their drinking within the past year, despite drinking less frequently than adults. Evidence suggests that younger drinkers are more vulnerable to acute alcohol-related harm due to lower alcohol tolerance, developmental vulnerability, and riskier drinking environments, [27] and experience disproportionately higher rates of unintentional injury and other acute harms following alcohol use [10,28]. In the Tanzanian context, this vulnerability may be amplified by misperceptions of drinking limits, poor coping mechanisms, and limited harm-reduction strategies among young people [5,29,30]. Economic disparities may also partly explain lower reported alcohol consumption among young people, [31] consistent with the lower income observed in this group.

Despite differences in drinking frequency and pattern, the comparable prevalence of AUD across young people and adults indicates that harmful alcohol use is not confined to one developmental stage. Notably, young people reported lower levels of alcohol consumption than adults, yet exhibited a similar prevalence of AUD, suggesting a disproportionate burden of harmful alcohol use relative to their level of exposure. This may reflect greater vulnerability to alcohol-related harm despite lower drinking frequency, consistent with developmental vulnerability and riskier drinking contexts in this age group [2]. Global burden analyses also identify alcohol as a leading risk factor for morbidity and mortality among adolescents and young people [32,33]. Importantly, the high prevalence of AUD observed among adults in this study is consistent with emerging evidence suggesting increasing rates of AUD in adult populations, [34] possibly related to shifting societal norms and reduced stigma around disclosure [35]. Together, these findings support routine AUD screening in emergency settings across age groups. However, alcohol-related harms may be more severe for young people, whose brains, particularly the prefrontal cortex, are still undergoing critical development. Given the similarly high AUD prevalence across age groups, early identification and intervention among young people remain particularly important to reduce long-term consequences.

Young people were significantly more likely than adults to report physical consequences of alcohol use, as captured by the DrInC physical subscale. These acute outcomes, including vomiting, blackouts, and alcohol-related injuries, are characteristic of early drinking trajectories, when tolerance and self-regulation are not yet established [36]. With prolonged exposure, adults may develop physiological tolerance or behavioural adaptations that reduce visible acute harm, although risk persists [37]. In Tanzania, habitual adult drinking patterns have been observed that may escape immediate clinical attention while contributing to long-term morbidity [30]. Although acute harms may be more visible among young people, chronic consequences among adults remain clinically important. This underscores the role of emergency departments as critical intervention points to interrupt the transition from early harmful alcohol use among young people to more established drinking patterns in adulthood.

### Limitations

This study has several limitations. The cross-sectional design limits causal inference between alcohol use patterns and injury outcomes. Alcohol use and consequences were self-reported, introducing potential recall and social desirability bias, particularly in a clinical setting. Although validated tools (AUDIT and DrInC) were used, underreporting or misclassification may have occurred. AUD was defined using AUDIT ≥8, which reflects hazardous or harmful use or probable dependence rather than a clinical diagnosis, potentially limiting comparability with studies using diagnostic criteria. The sample included only injury patients who were clinically sober and able to consent, potentially excluding those with severe intoxication or cognitive impairment and thus underestimating the true burden of alcohol-related harm. As a secondary analysis of registry and trial data, available variables were limited, and analyses were descriptive without adjustment for potential confounders such as sex, income, and injury type. Findings are based on a single referral hospital in Northern Tanzania, which may limit generalizability. However, the hospital serves a wide urban and rural catchment area, suggesting reasonable representation of diverse patient experiences.

## Conclusion

This study highlights age-related differences in alcohol use patterns and alcohol-related harms among injury patients in Northern Tanzania. While adults reported more frequent alcohol consumption, young people experienced higher alcohol-related physical consequences and were more likely to report injury resulting from their drinking. Despite these differences, both groups showed similarly high prevalence of alcohol use disorder, underscoring the widespread nature of harmful drinking in this population. These findings support age-tailored prevention and response strategies within emergency care and public health systems, particularly approaches targeting risky drinking behaviours among young people and sustained use among adults. Future research should evaluate age-response adaptations of existing brief alcohol interventions in emergency settings and examine longitudinal pathways linking alcohol use and injury risk in resource-limited settings.

## Funding

This study was supported by the Trauma Research Capacity Building Program at KCMC through the NIH D43 TRECK grant (D43-TW012205) and the Duke Department of Emergency Medicine (PIs: Mmbaga and Staton). WM and BM were supported by the NIH-funded D43 TRECK program. The PRACT Trial was supported by the National Institute on Alcohol Abuse and Alcoholism (NIAAA), NIH (1R01AA027512-01A1; PI: Staton). The funders had no role in study design, data collection, analysis, interpretation, or manuscript preparation.

## Dissemination of results

Findings from this study were presented to the GEMINI research group, the KCMC University PhD Symposium, and the Duke Global Mental Health Conference 2025 to facilitate academic feedback and knowledge exchange.

## CRediT authorship contribution statement

**Winfrida C. Mwita:** Conceptualization, Methodology, Formal analysis, Visualization, Writing – original draft. **Elizabeth M. Keating:** Validation, Writing – review & editing. **Rune Nathaniel Philemon:** Conceptualization, Methodology, Writing – review & editing. **Baraka Moshi:** Validation, Writing – review & editing. **Doreen O. Msemakweli:** Validation, Writing – review & editing. **Alice Andongolile:** Validation, Writing – review & editing. **Florida J. Muro:** Conceptualization, Methodology, Supervision, Writing – review & editing. **Blandina T. Mmbaga:** Validation, Writing – review & editing. **João Ricardo Nickenig Vissoci:** Conceptualization, Methodology, Investigation, Supervision, Funding acquisition, Writing – review & editing. **Sia E. Msuya:** Conceptualization, Methodology, Supervision, Writing – review & editing. **Catherine A. Staton:** Conceptualization, Methodology, Investigation, Supervision, Funding acquisition, Writing – review & editing.

## Declaration of competing interest

We have no conflict of interest to declare
